# Comparison of herbal medicines and pain relief medications in the treatment of primary dysmenorrhoea among female medical students at Taibah University

**DOI:** 10.1016/j.jtumed.2022.10.015

**Published:** 2022-11-12

**Authors:** Amal Yaseen Zaman, Afrah M. Alameen, Mawadah M. Alreefi, Sarah T. Kashkari, Samaher A. Alnajdi, Afkar A. Shararah, Sarah M. Alzolaibani, Fai A. Mahrous

**Affiliations:** a Department of Obstetrics and Gynecology, Faculty of Medicine, Taibah University, Almadinah Almunawwarah, KSA; b Faculty of Medicine, Taibah University, Almadinah Almunawwarah, KSA

**Keywords:** أنشطة الحياة اليومية, طب الأعشاب, مسكنات الألم, انتشار, عسر الطمث الأولي, جامعة طيبة, Daily life activities, Herbal medicine, Pain relief medications, Prevalence, Primary dysmenorrhoea, Taibah University

## Abstract

**Objectives:**

Dysmenorrhoea is a common gynaecological problem that affects many women during their reproductive years. The objectives of this study were to describe the different treatments used for primary dysmenorrhoea (PD) among medical students at Taibah University, and to investigate the link between pain severity and daily life activities in relation to the type of dysmenorrhoea treatment.

**Methods:**

A cross-sectional study was conducted on 301 female medical students through an 18-item self-administered electronic questionnaire to screen for students with PD. The questionnaire included sociodemographic characteristics, details of self-management methods (types and adverse events), daily life domains affected by pain, and the Visual Analogue Scale score for the pain (wherein a score ≥7 indicated severe pain). Chi-square test, a multivariate regression model, and correlation analysis were used for data analysis.

**Results:**

The prevalence of PD among respondents was 71.8%. Medications were used by more than half of the respondents (51.9%), and were mainly non-steroidal anti-inflammatory drugs (53.5%) and paracetamol (47.5%). Among the participants, 14.1% used herbal medicines, cinnamon (55.7%), chamomile (40.7%), and ginger (33.3%). Other pain relief modalities were used by 34% of participants. Most students with a history of PD (80.6%) reported effects on their daily activities, mainly mood disturbance. Students using medications were more likely to have severe pain (72.7%) and an affect on daily activities (92.9%) than those using herbal medicines (44.4% and 88.9%, respectively) and other treatments (47.7% and 70.8%, respectively) (p < 0.001).

**Conclusions:**

Medications were more commonly used than herbal medicines and other relief methods. Effects of PD on daily life activities were observed among most students treated with medications. We recommend health promotion programmes to increase the awareness regarding different pain relief methods.

## الملخص

**أهداف البحث:**

عسر الطمث هو مشكلة نسائية شائعة تصيب العديد من الإناث خلال سنوات الإنجاب. لذلك أردنا أن تكون أهداف هذه الدراسة: وصف العلاجات المختلفة المستخدمة لعسر الطمث الأولي بين طالبات الطب في جامعة طيبة والتحقق من العلاقة بين شدة الألم وأنشطة الحياة اليومية فيما يتعلق بنوع علاج عسر الطمث.

**طريقة البحث:**

أجريت دراسة مقطعية على 301 طالبة طب باستخدام استبانة إلكترونية مكونه من 18 بندًا من أجل فحص الطالبات اللواتي يعانين من عسر الطمث الأولي. تضمنت الاستبانة الخصائص الاجتماعية الديموغرافية، وتفاصيل طرق العلاج المختلفة وأعراضها الجانبية، ومجالات الحياة اليومية المتأثرة بالألم، والمقياس المرئي للألم; علماً بأن الدرجة ≥ 7 في المقياس المرئي تُمثل ألمًا شديدًا. وقد تم استخدام اختبار مربع كاي -اختبار احصائي يساعد على معرفة مدى التطابق-. ونموذج الانحدار متعدد المتغيرات وتحليل الارتباط لتحليل البيانات.

**النتائج:**

كان معدل انتشار عسر الطمث الأولي بين المشاركات 71.8٪. حيث وجد أن 51.9٪ استخدمن مسكنات الألم، وكان أكثرها استخداماً هي مضادات الالتهاب الغير ستيرويدية (53.5٪) والباراسيتامول بنسبة 47.5٪ بينما 14.1٪ من المشاركات استخدمن الأدوية العشبية مثل القرفة (55.7٪) والبابونج (40.7٪) والزنجبيل (33.3٪). والبقية بنسبة 34٪ من المشاركات قمن باستخدام طرق تخفيف أخرى. وبالنسبة لتأثير عسر الطمث الأولي على المشاركات; فقد أكّد 80.6٪ على تأثير ذلك على أنشطتهم اليومية، وخاصتاً اضطراب المزاج. واتضح أن اللواتي يستخدمن الأدوية كُن الأكثر إصابة بآلام شديدة (72.7٪) والأكثر تأثراً على الأنشطة اليومية (92.9٪) مقارنةً مع اللواتي عولِجن بالأعشاب (44.4٪ و88.9٪ على التوالي) والعلاجات الأخرى (47.7٪ و70.8٪ على التوالي).

**الاستنتاجات:**

خلاصة دراستنا، تبيّن أن مسكنات الألم تُعد أكثر استخداماً مقارنةً بالأدوية العشبية وطرق التخفيف الأخرى. لوحظ أن تأثير عسر الطمث الأولي على أنشطة الحياة اليومية لدى معظم الطالبات اللواتي عولِجن بالأدوية. وفي الختام نوصِي برامج تعزيز الصحة بزيادة الوعي بالأساليب المختلفة لتخفيف الآلام.

## Introduction

Dysmenorrhoea is a common gynaecological disorder affecting many women during their reproductive years.^1^ This condition is defined as a painful, cramping sensation in the lower abdomen; it is directly associated with menstruation, and its symptoms include headache, backache, leg pain, diarrhoea, and nausea.^2^ Dysmenorrhoea is divided into primary and secondary types on the basis of pathophysiology. Primary dysmenorrhoea (PD) is a spasmodic lower abdominal pain that occurs just before and/or during menstruation in the absence of any pelvic or hormonal pathology. In comparison, secondary dysmenorrhoeic pain may originate from various pathological conditions, such as endometriosis, adenomyosis, fibroids, and pelvic inflammatory disease.^1^^,^^3^ In women from different countries in different age groups, the prevalence of PD has been estimated to be 45.0–94.4%.^4^, ^5^, ^6^, ^7^ A Mexican study has reported that 10% of women may have severe dysmenorrhoea that disturbs their daily activities.^8^ In Kingdom of Saudi Arabia (KSA), a cross-sectional study performed in 2015 at King Abdulaziz University has found a high prevalence of dysmenorrhoea among medical students (60.9%).^9^ Although dysmenorrhea is a substantial health problem, no studies have been conducted on this important problem among medical students in Almadinah Almunawwarah.

Prostaglandins (PGs) play a major role in the pathophysiology of PD. The increase in PG release is responsible for the contraction of uterine smooth muscles, which causes the lower abdominal and back pain characteristic of dysmenorrhoea. Similarly, PGs are responsible for the smooth muscle contraction of the gastrointestinal tract, and consequently can lead to many symptoms such as nausea, vomiting, and diarrhoea.^10^, ^11^, ^12^, ^13^ Patients with dysmenorrhoea with higher levels of PGs have higher contraction rates.^14^

The management of dysmenorrhoea is based mainly on two strategies: pharmacological and non-pharmacological treatment.^15^ Non-steroidal anti-inflammatory drugs (NSAIDs), such as ibuprofen, naproxen, and mefenamic acid, are usually used in women with PD.^16^ Other drugs, such as oral contraceptive pills (OCPs), antispasmodic medications, and acetaminophen, are also used. However, long-term use of NSAIDs and OCPs may have adverse effects, such as injury to the gastrointestinal tract mucosa, thus leading to GI discomfort.^17^ The adverse effects associated with such treatments have led women to seek complementary and alternative medicine treatments, such as using various herbs, food, or exercise. Many physicians worldwide have used medicinal plants, such as fennel, ginger, and cinnamon, because of their antinociceptive effects.^17^^,^^18^ Moreover, local heat, exercise, rest, and fatty diet restriction have shown beneficial effects in the management of PD, and no severe adverse effects have been reported.^2^^,^^16^^,^^19^ Despite the adverse effects of pharmacological treatments, they are still considered the most effective and reliable treatments for dysmenorrhoea.^20^

To our knowledge, many studies have reported PD prevalence and self-care strategies. However, in KSA, specifically in Almadinah Almunawwarah, no studies have been published on the use of herbal medicines among women with dysmenorrhoea during the time interval from 2010 to 2020, according to the PubMed and Cochrane databases. This study therefore aimed to describe the different treatments used for PD among medical students. In addition, we aimed to investigate the link between pain severity and daily life activities in relation to the type of dysmenorrhea treatment.

## Materials and Methods

### Study design and setting

This was a cross-sectional study at Taibah University in Almadinah Almunawwarah, KSA, which was performed during the period of October 2020–December 2021.

### Study population and sampling

This study included medical students in their first to fifth years. Participants who did not consent to fill the questionnaire were excluded. A convenience sampling method was used to select the sampling units. The sampling fraction was 301 of 386 medical students, which comprised 77.97% of all female medical students at Taibah University. The sample size was calculated with the Open epi software calculator, and the minimum sample size was 193.

### Study measurements

Data collection was performed with an anonymous English and Arabic self-administered electronic questionnaire containing 18 items. The variables in the questionnaire included socio-demographic information (age, medical year, and marital status), health status, details of PD, such as severity of pain, and pain management strategies (either analgesics or herbal medicines)—the cornerstone of our study. The questionnaire also included other methods for managing dysmenorrhoea, such as exercise, applying heat packs, and rest, and how these strategies affected the students’ daily life activities (Appendices).

### Data analysis

Data collection and entry were performed in Microsoft Excel version 16.0.1. Data analysis was performed in Statistical Product and Service Solutions (SPSS) version V26. For the descriptive analysis and qualitative variables, frequencies and percentages were used. The Visual Analogue Scale was used for dysmenorrhoeic pain, on a scale of 1–10. A score ≤3 was considered to indicate mild pain, a score between 4 and 6 was considered to indicate moderate pain, and a score ≥7 was considered to indicate severe pain. To determine the significant associations among variables, a chi-square test was used, and a P-value < 0.05 among different variables was considered statistically significant. Multivariate regression model and correlation analysis were also used for data analysis.

Participation of students in the study was based on an informed consent option chosen before completion of the questionnaire. All participants were assured that all data collected would be confidential and would not be used for any purposes except the study. This study was ethically approved by the Research Ethical Committee of Taibah University in Almadinah Almunawwarah, KSA, in Decem-ber of 2020, with approval No. 20-004.

## Results

The study included 301 female medical students. Their personal characteristics are summarised in Table 1. A total of 194 students (64.5%) were 21–23 years of age, and 7.3% (*n* = 22) were older than 23 years of age. The highest proportion of participants was recruited from students in their third academic year (74; 24.5%), whereas the lowest proportion of participants was recruited from the fifth academic year (42; 14%). Most students (290; 96.3%) were single. A history of chronic diseases was observed among 5.3% (*n* = 16) of participants.

**Table 1:** Personal characteristics of the participants (n = 301).

	Frequency	Percentage
***Age (years)***		
18–20	85	28.2
21–23	194	64.5
≥ 24	22	7.3
***Medical year***		
First	60	19.9
Second	58	19.3
Third	74	24.5
Fourth	67	22.3
Fifth	42	14.0
***Marital status***		
Single	290	96.3
Married	11	3.7
***History of chronic medical illnesses***		
Yes	16	5.3
No	285	94.7

The prevalence of PD among respondents was 71.8% (*n* = 216). More than half the students with PD (122; 56.5%) described their pain as severe. Most students with PD (191; 88.4%) reported a history of using different methods to relieve the pain. Medications were used by more than half the participants (99; 51.9%); mainly NSAIDs (53; 53.5%) and paracetamol, (47; 47.5%). Adverse effects due to medication use were reported in 8.1% (*n* = 8) of the students who used them, and these effects consisted mainly of vomiting (6; 62.5%), nausea (5; 50%), and diarrhoea (5; 50%).

Herbal medicines were used to relieve PD pain by 14.1% (*n* = 27) of the students. Commonly used herbal medicines were cinnamon (15; 55.7%), chamomile (11; 40.7%), and ginger (9; 33.3%).

Most students with a history of PD reported that their daily activities were affected (174; 80.6%). The reported effects were mood disturbance (31; 17.8%), decreased concentration and academic performance (11; 6.3%), sleep deprivation (10; 5.7%), and college absence (3; 1.7%). Moreover, 48.3% (*n* = 84) of the cohort reported three or more daily activity domains affected (Table 2).

**Table 2:** Daily activity domains affected among the medical students (n = 174).

Q: How was your daily activity affected? (In this question, the student was able to choose more than one choice)	Number	Percentage
Mood disturbance	31	17.8%
Decreased concentration and academic performance	11	6.3%
Sleep deprivation	10	5.7%
College absence	3	1.7%
Two domains affected	35	20.1%
Three or all domains affected	84	48.3%
Total	174	100%

Severe pain was observed among most students treated with medications (72; 72.7%), compared with 44.4% (*n* = 12) and 47.7% (*n* = 31), who were treated with herbal medicines and other methods, respectively (p = 0.001). Similarly, daily activities were affected in most students treated with medications (92; 92.9%), compared with 88.9% (*n* = 24) and 70.8% (*n* = 46) of those treated by herbal medicines and other methods, respectively (p < 0.001) (Table 3).

**Table 3:** Comparison among modes of primary dysmenorrhoea treatment in medical students (n = 191).

	Medications n = 99	Herbal medicines n = 27	Others∗ n = 65	P-value∗∗
	n (%)	n (%)	n (%)	
***Pain severity***				
Mild (n = 10)	1 (1.0)	4 (14.8)	5 (7.7)	
Moderate (n = 66)	26 (26.3)	11 (40.7)	29 (44.6)	
Severe (n = 115)	72 (72.7)	12 (44.4)	31 (47.7)	0.001
***Affected daily activities***				
No (n = 29)	7 (7.1)	3 (11.1)	19 (29.2)	
Yes (n = 162)	92 (92.9)	24 (88.9)	46 (70.8)	<0.001

∗ rest, exercise, heat packs.

∗∗ chi-square test.

In the logistic regression model, increasing menstrual pain severity was a predictor of daily activities affected (OR and 95% CI 1.49 (1.23–1.79), p < 0.001), as shown in Table 4.

**Table 4:** Relationships of primary dysmenorrhoea with age, medical year, and pain severity.

Variable	OR	95% CI	P value∗
Age	0.58	0.088–3.89	0.07
Medical year	1.18	0.78–1.79	0.42
Pain severity	1.49	1.23–1.79	<0.001

∗ logistic regression test.

## Discussion

Our study indicated that the prevalence of PD was 71.8%. Medications were the main method used among participants (51.9%), followed by other relief modalities and herbal medicines (34% and 14.1%, respectively). Most students with a history of PD (80.6%) reported effects on their daily activities. The use of medication was significantly associated with reporting of severe pain and effects on daily life activities. Moreover, higher menstrual pain severity was found to be a predictor of effects on daily activities (p < 0.001).

In the current study, among 301 participants, 71.8% had dysmenorrhoea, a percentage similar to that reported a study in the southern region of KSA (70.6%). Unexpectedly, high variation has been observed in the prevalence rates among regions of the KSA, despite being in the same country. Specifically, a severe dysmenorrhoea rate of 87.7% has been reported in the northern region, 60.9% has been reported in the western region, and 35% has been reported in the eastern region.^9^^,^^20^, ^21^, ^22^ Generally, global variations in the prevalence rate have been documented, ranging from 51.1% to 98.4%.^2^^,^^23^, ^24^, ^25^ In previous studies, the variations in prevalence rates of dysmenorrhoea have been attributed to the lack of an accepted universal method for defining dysmenorrhoea.^20^ Moreover, the target populations among the studies differed in age and culture.

Pain is a subjective symptom and is therefore difficult to estimate. Accordingly, researchers have established various scoring systems to measure pain severity.^26^

We used the Visual Analogue Scale, with scores from 1 to 10; any score ≤3 was considered to indicate mild pain, any score between 4 and 6 was considered to indicate moderate pain, and any score ≥7 was considered to indicate severe pain. Almost 56.5% of the participants had severe pain, whereas only 6.9% had mild pain. Our results regarding pain severity were relatively similar to those in studies performed in Iran, Spain, and various regions in KSA,^9^^,^^19^^,^^25^ whereas in some studies, most participants classified their pain as moderate.^20^

The degree of pain may vary because of familial, genetic, psychosocial, and cultural factors.^2^ In addition, the different sample sizes and scales used might have affected the results.^9^ All these factors may substantially account for pain perception and the variability in pain thresholds.

Both the severity of pain and the absence of appropriate relief methods have been reported to markedly affect women's daily routines and limit their activities.^25^ However, our study found a significant association between the pain intensity of dysmenorrhoea and the use of different methods to relieve pain (p = 0.001). Similarly, a study in Ghana has found that women with more severe dysmenorrhoea exhibit a significantly greater tendency to seek treatment (p < 0.0001).^2^

The results of this study showed that most students (88.4%) reported a history of using pain relieving methods, in accordance with findings from a study performed in India, in which 86.9% of respondents sought treatment.^27^ According to the previous study, the most common relief method was the application of heat packs, whereas in our study, the most common method used to relieve dysmenorrhoeal pain was medications (51.9%), mainly NSAIDs. In addition, NSAIDs have been the most used method in many studies.^2^^,^^3^^,^^19^^,^^20^^,^^25^^,^^26^ Anti-inflammatory drugs, such as NSAIDs, antagonize the effects of PGs, which are considered the source of dysmenorrhoeal pain.^28^ Despite their effectiveness, NSAIDs are not commonly used in Hong Kong, probably because they are prescription drugs and are associated with adverse effects.^24^ However, in a study conducted in Jordan among female medical students, the most used medication to relieve menstrual pain was paracetamol (61.2%).^26^ The use of paracetamol for the management of dysmenorrhoea has also been reported in previous studies.^14^^,^^24^ Paracetamol, although it exhibits a weaker analgesic effect than NSAIDs, is better tolerated and has a better safety profile.^29^

Doaa et al. have reported that herbs were the main form of treatment used among their study participants (60.1%).^21^ However, this result is not in accordance with our results, in which herbs were used among only 14.1% of our study population, and cinnamon was the most used (55.7%). Cinnamon extract (eugenol) can prevent the biosynthesis of PGs and decrease inflammation.^18^ Ginger was also used among 33.3% of the participants in our study. A clinical trial in Iran has found that ginger is as effective as ibuprofen in relieving pain in women with PD, because it is a potent inhibitor of PG (via cyclooxygenase and lipoxygenase inhibition), and is used as a traditional remedy for treating dysmenorrhoea.^30^ Furthermore, fennel was used among only 14.8% of our participants. Studies have found that fennel has anti-inflammatory, analgesic, and antispasmodic effects, and is effective in decreasing the severity of dysmenorrhoea.^19^, ^20^, ^21^^,^^25^, ^26^, ^27^^,^^29^ One reason for variations in choosing the management methods of PD is cultural background; for example, traditional Chinese medicine was widely trusted by Chinese girls in regard to pharmacological treatment.^23^

In the present study, most students with PD reported an effect on daily activities (80.6%), in which mood disturbance was the main effect (81%). This finding is comparable to those from a study conducted in Hong Kong, in which concentration disturbance was the main effect (75%).^24^ However, our results indicated that attending college was the activity least affected, in agreement with findings from studies in the KSA and Iran.^9^^,^^25^ In contrast, a Ghanaian study has reported that 70% of participants indicated that lecture attendance was the most affected activity.^2^

Asvini et al. have found a significant association between limitations in daily life activities and severity of dysmenorrhoea.^3^ In the present study, however, the effect on daily life activity was significantly associated with the use of medication. In summary, PD poses a major strain not only on students but also on the quality of their learning process.

### Study strengths and limitations

A major strength of this study is that it reports what is, to our knowledge, the first investigation of the prevalence of the use of various modalities for relieving PD and the effect of dysmenorrhoea on medical students' daily lives in Almadinah Almunawwarah. However, the study has several limitations. First, the study sampling method (convenience sampling) and the inclusion of only medical students may limit the generalizability of the results. Being a medical student and receiving training in different related subjects (e.g. pharmacology) was likely to have affected the choice of dysmenorrhoea treatment and the ability to self-medicate. Second, we could not validate our study questionnaire, because the circumstances of application of the questionnaire method did not allow us to proceed with the first step, i.e., validation. Third, although 80% of female medical students participated in the study, the sample was obtained from one university in one region of the KSA. Therefore, studies from different university populations should be examined to include different traditions and weather conditions among regions of KSA, which might affect the results. Despite these limitations, this study provides useful information regarding menstrual health care issues among Saudi university students and may provide insights for future research in this field to learn more about this area and address the aforementioned issues.

## Conclusions and recommendations

In this study, we found a high prevalence of dysmenorrhoea among female medical students at Taibah University. More than half the participants used medications, specifically NSAIDs, rather than herbal medicine. The use of medication was significantly associated with reporting of severe pain and effects on daily life activities. Because PD affected daily life activities in more than 80% of the participants, we recommend health promotion programmes to increase the awareness of different pain-relieving methods. Future research may focus on the effectiveness of pharmacological and non-pharmacological management strategies.

## Source of funding

This research did not receive any specific grant from funding agencies in the public, commercial, or not-for-profit sectors.

## Conflict of interest

The authors have no conflicts of interest to declare.

## Ethical approval

At the beginning of the questionnaire, a brief definition of PD was included for clarification purposes. The students were informed of the purpose of our study and encouraged to share their experiences by completing the electronic questionnaire. Participation of the students in the study was based on an informed consent option to choose prior to completing the questionnaire. All participants were assured that all data collected will be confidential and not be used for any purposes except the study. This study was ethically approved by the Research Ethical Committee of Taibah University in Almadinah Almunawwarah, KSA, in December of 2020, with approval No. 20–004.

## Authors contributions

All authors participated equally in conceiving and designing the study; conducting research; providing research materials; collecting and organizing data; analysing and interpreting data; writing the initial and final draft of the article; and providing logistic support. All authors have critically reviewed and approved the final draft and are responsible for the content and similarity index of the manuscript.

## Footnotes

Appendix A Supplementary data to this article can be found online at https://doi.org/10.1016/j.jtumed.2022.10.015.
