# Supplementary figures and images for: Comparison of herbal medicines and pain relief medications in the treatment of primary dysmenorrhoea among female medical students at Taibah University
Source: J Taibah Univ Med Sci. 2022 Nov 12;18(3):455–60. doi: 10.1016/j.jtumed.2022.10.015 (PMC9932468; doi:10.1016/j.jtumed.2022.10.015)

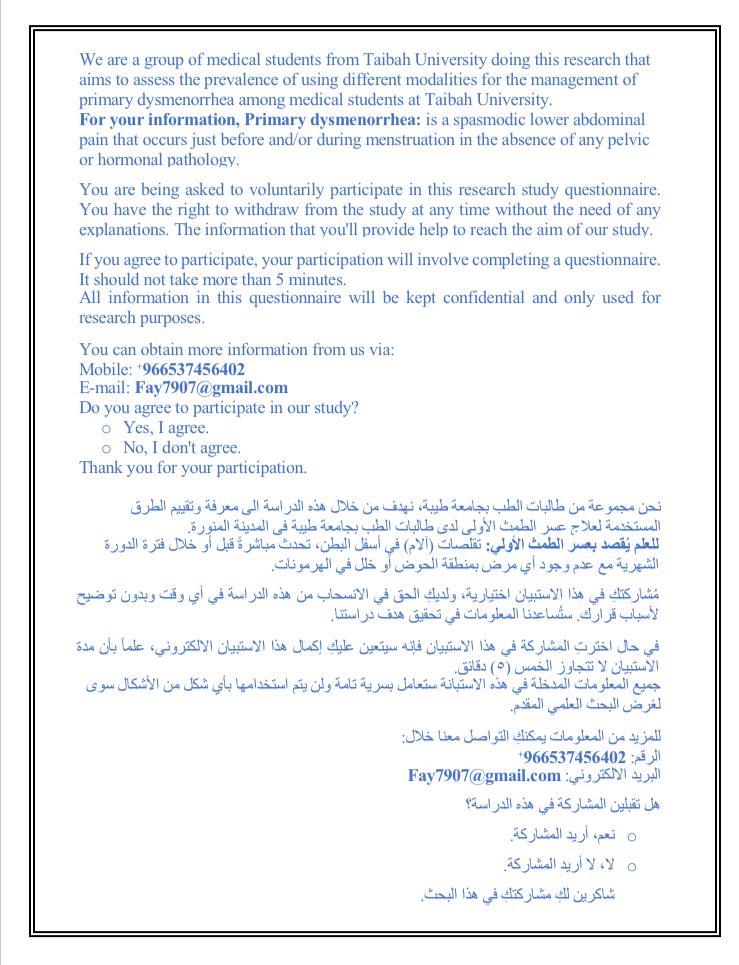
**Appendices**

**
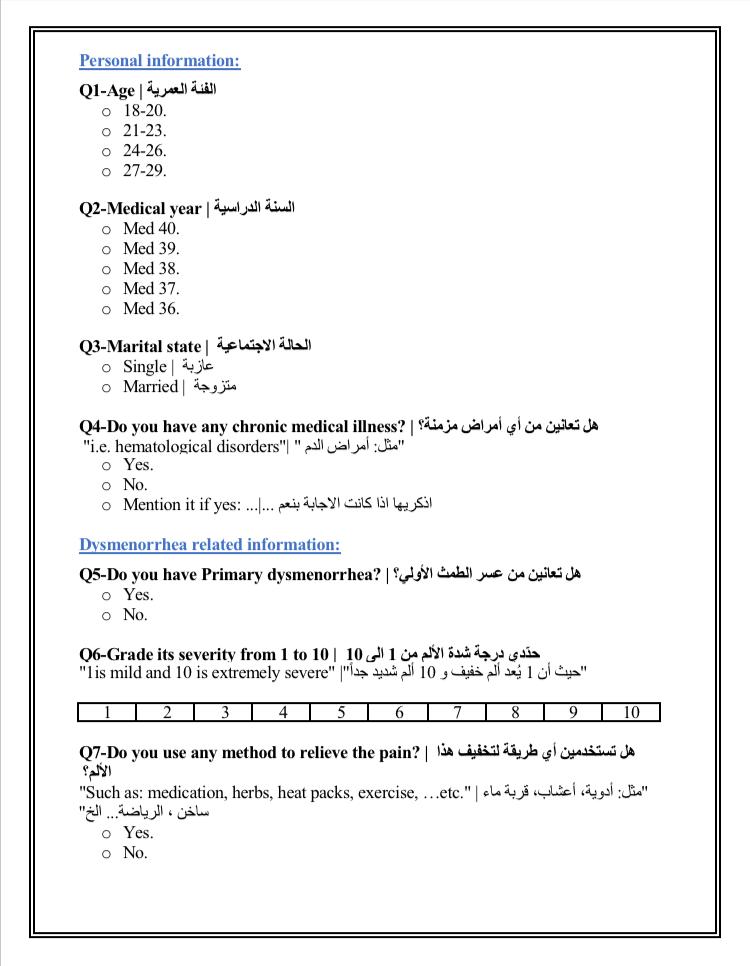
Appendix** (continued)

**
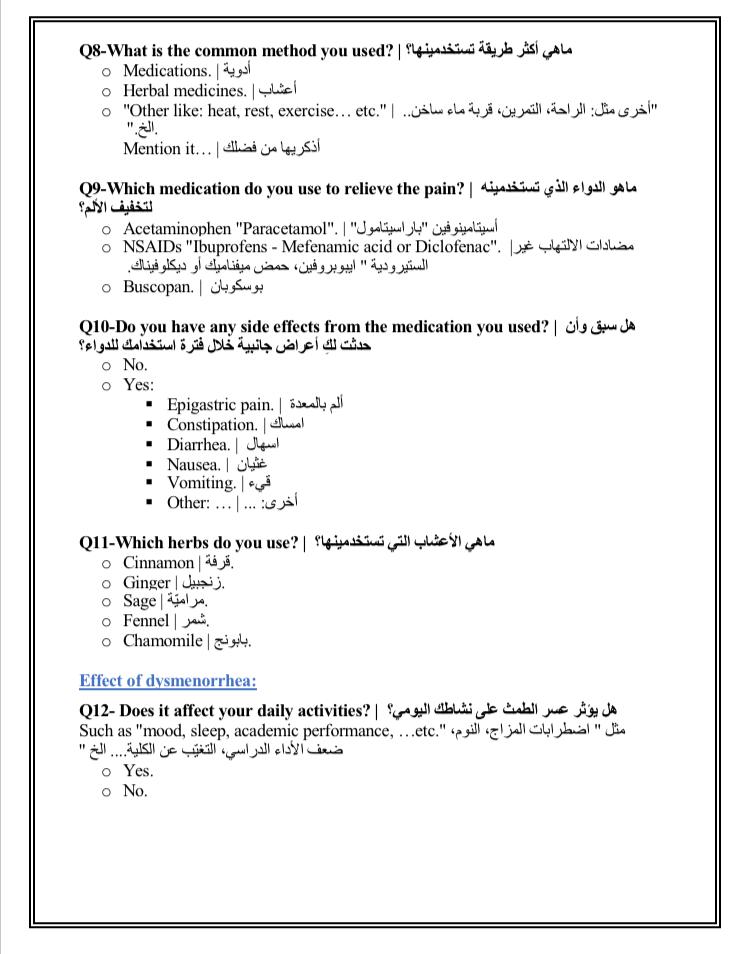
Appendix** (continued)

**
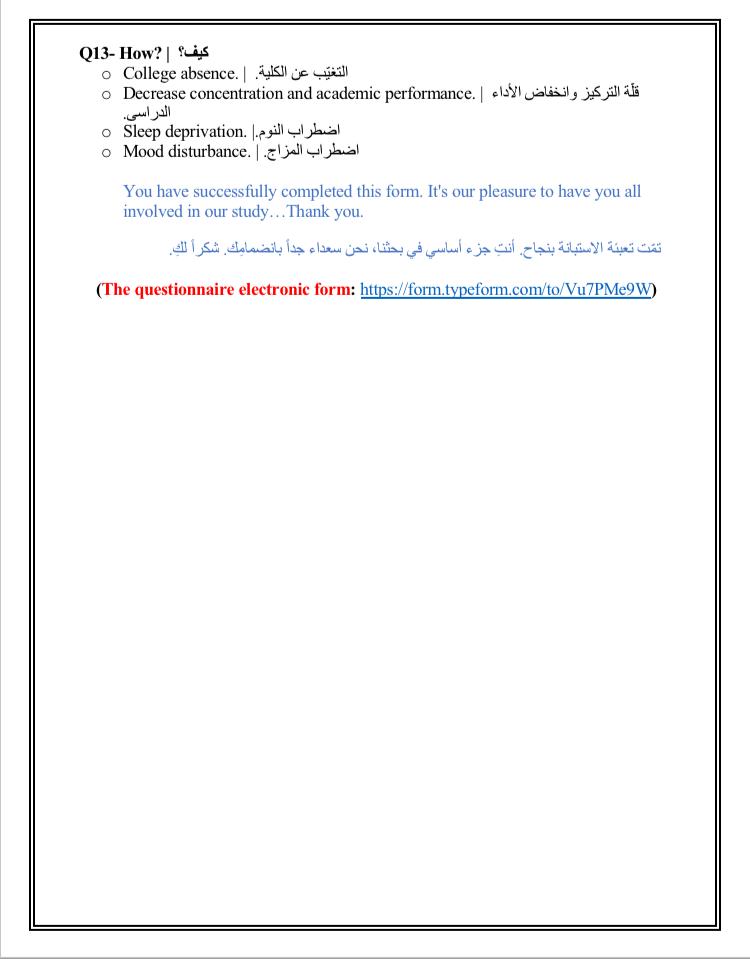
Appendix** (continued)

Supplement: Multimedia component 1 [file mmc1.docx]
